# Construction and Comprehensive Prognostic Analysis of a Novel Immune-Related lncRNA Signature and Immune Landscape in Gastric Cancer

**DOI:** 10.1155/2022/4105280

**Published:** 2022-01-17

**Authors:** Xiaolong Liang, Lang Zha, Gangfeng Yu, Xiong Guo, Chuan Qin, Anqi Cheng, Ziwei Wang

**Affiliations:** ^1^Department of Gastrointestinal Surgery, The First Affiliated Hospital of Chongqing Medical University, Chongqing 400010, China; ^2^Institute of Life Sciences, Chongqing Medical University, Chongqing 400010, China

## Abstract

Gastric cancer (GC) is a malignant tumor with high mortality and poor prognosis. Immunotherapies, especially immune checkpoint inhibitors (ICI), are widely used in various tumors, but patients with GC do not benefit much from immunotherapies. Therefore, effective predictive biomarkers are urgently needed for GC patients to realize the benefits of immunotherapy. Recent studies have indicated that long noncoding RNAs (lncRNAs) could be used as biomarkers in the immune landscape of multiple tumors. In this study, we constructed a novel immune-related lncRNA (irlncRNA) risk model to predict the survival and immune landscape of GC patients. First, we identified differentially expressed irlncRNAs (DEirlncRNAs) from RNA-Seq data of The Cancer Genome Atlas (TCGA). By using various algorithms, we constructed a risk model with 11 DEirlncRNA pairs. We then tested the accuracy of the risk model, demonstrating that the risk model has good efficiency in predicting the prognosis of GC patients. Inner validation sets were further used to confirm the effectiveness of the risk model. In addition, our risk model has a preferable performance in predicting the immune infiltration status of tumors, immune checkpoint status of the patients, and immunotherapy score. In conclusion, our risk model may provide insights into the prognosis of and immunotherapy strategy for GC.

## 1. Introduction

Gastric cancer (GC), one of the most common malignant tumors globally, ranks as the fifth and fourth most common cause of cancer incidence and mortality, respectively [1]. The morbidity of GC has decreased year by year in many areas, but the incidence of gastric cancer in Asia, especially in eastern Asia, remains the highest [1]. Although great progress has been made in the diagnosis and treatment of GC in the last decade, the overall survival of patients after treatment with conventional first-line chemotherapy and second-line chemotherapy remains poor, especially given that the median survival of advanced gastric cancer (AGC) is less than one year [2].

In recent years, increasing research on immunity, immunotherapy, and immune checkpoint-related treatment has made effective treatment possible [3]. Immunotherapy prolongs the overall survival (OS) of patients with a variety of cancers [4–7]. Most types of tumors that benefit from immunotherapy have increased somatic mutations [8]. Patients with GC also have a higher somatic mutation rate, indicating that immunotherapy might be effective for GC patients [9]. At present, immunotherapy for gastric cancer mainly includes anti-CTLA-4, anti-PD-1/PD-L1, or the combination of anti-CTLA-4 and anti-PD-1 antibodies [10]. A phase II clinical study including 18 patients has indicated that tremelimumab, a monoclonal antibody targeting CTLA-4, is effective in the treatment of late-stage GC [11]. However, another phase II clinical study has revealed that ipilimumab (another inhibitor of CTLA-4) does not prolong the OS of patients [12]. Several clinical trials have reported that anti-PD-1 monoclonal antibodies prolong the OS of GC patients [13, 14]. However, another phase III trial has demonstrated that pembrolizumab does not significantly prolong the OS of patients with GC [15]. Immunotherapy is not effective for all patients; it is only for some patients. Therefore, it is of great significance to select the proper patients to receive immune therapy. At present, the role and type of immune landscape in the prognosis of GC remain largely unknown. Identification of infiltrating immune cells is associated with cancer prognosis and new immune therapeutic targets, which might provide meaningful clues for the future treatment of gastric cancer, especially for immunotherapy.

lncRNAs are defined as noncoding RNAs with a length greater than 200 nucleotides [16]. lncRNAs account for up to 97% of RNA in the cell and were once considered as insignificant “noise” [17, 18]. Recent studies have revealed that various lncRNAs are abnormally expressed in many cancers and regulate numerous biological processes associated with tumorigenesis [19–22]. Some lncRNAs have been identified as potential biomarkers for diagnosis, prognosis, and therapeutic targets in multiple human cancers [23–25]. Moreover, lncRNAs have been reported to play vital roles in cancer immunity [26]. Recent evidence has demonstrated that lncRNAs could also be used as biomarkers in predicting the immune landscape of multiple cancers [27, 28]. However, the correlation between lncRNAs and the immune landscape in GC remains largely unknown.

In the present study, we constructed a novel prognostic model based on immune-related lncRNA (irlncRNA) pairs. Compared with the single-gene prognostic model, the two-biomarker combination prognostic model is superior in predicting the survival of GC patients obtained from the TCGA databases. In addition, we explored the relationship between the risk model and immune cell infiltration, immune checkpoints, and immunotherapy. We demonstrated that our model shows advanced efficiency in predicting the survival of patients, the infiltration of immune cells, and the effectiveness of immunotherapy.

## 2. Materials and Methods

### 2.1. Data Acquisition, Processing, and Differentially Expressed Analysis

The transcriptome data of gastric cancer (GC) and matched clinical information were obtained from TCGA-STAD project (https://tcga-data.nci.nih.gov/tcga/). Patients with survived less than 30 days and incomplete clinical information were excluded. The human GTF file was downloaded from Ensembl (http://asia.ensembl.org) and used to obtain lncRNA expression data from transcriptome data. The certified immune-related genes in the ImmPort database (http://www.immport.org) were obtained and used to identify immune related lncRNAs (irlncRNAs) by using coexpression strategy. We used 1706 certified immune-related genes to identify immune-related lncRNAs (irlncRNAs) using a screening based on Pearson's correlation analysis (threshold of ∣*R*2 | >0.4; *P* < 0.001). Then, limma package of R software was utilized to screen differential irlncRNAs (DEirlncRNAs) between tumor tissue and adjacent normal tissues (FC > 2, *P* < 0.05). This study was approved by the Ethics Committee of the first affiliated hospital of Chongqing medical university.

### 2.2. Definition of Pairing DEirlncRNAs

The DEirlncRNAs were cyclically paired (such as lncRNA A and lncRNA B) to construct a new matrix. In this matrix, the value of each pair is calculated as follows: if the expression level of lncRNA A is higher than lncRNA B, the value of this lncRNA pair (*V*) will be defined as *V* = 1; if the expression level of lncRNA A is lower than lncRNA B, the value of this lncRNA pair will be defined as *V* = 0. If the *V* value of a paired lncRNA is 0 or 1 in more than 80% of the samples, this paired lncRNA will be removed. The rest lncRNA pairs were considered as valid match and used for further analysis.

### 2.3. Construction and Prognosis of the Risk Model

We performed univariate analysis to identify prognostic lncRNA pairs. Then, the least absolute shrinkage selection operator (LASSO) was conducted for 1000 cycles to acquire risk model (frequency more than 100 times). Next, the lncRNA pairs in risk model were used for the Cox regression analysis to acquire the risk score. The calculation of risk score is as follows: Risk score(patients) = ∑_*i*=1_^*K*^ *βi*∗*S* *i*. The accuracy of the prognostic model was assessed by a time-dependent receiver operating characteristic (ROC). The maximum inflection point of the ROC curve was identified and considered as the cut-off point to distinguished high or low-risk patients. By using survival package and survminer package of R software, we performed Kaplan-Meier analysis to visualize the survival difference between the high-risk group and the low-risk group.

### 2.4. Validation of the Risk Model

To further verify the accuracy of our risk model, we randomly divided all samples into two sets: validation set 1 and validation set 2 at a ratio of 5 : 5. A total of 153 samples and 152 samples were enrolled into validation set 1 and validation set 2, respectively. By using time-dependent ROC curve, we validated the accuracy of our risk model in two validation sets. The maximum inflection point of the ROC curve was identified and visualized. The survival status of high-risk group patients and low-risk group patients was visualized using Kaplan-Meier curve.

### 2.5. Clinical Application Value of the Risk Model

A chi-square test was performed to analyze the relationship between the risk model and clinicopathological characteristics. Univariate analysis was used to identify whether risk score and clinical features were associated with the prognosis of patients. Multivariate analysis was used to analyze whether risk score has independent prognostic function. ROC curve and decision curve analysis (DCA) were performed to validate the clinical application accuracy of the risk model. In addition, the “regplot” and “survival” R packages were used to construct a nomogram for predicting survival time of patients. The calibration curve was plotted to assess the accuracy of the nomogram.

### 2.6. Correlation between the Risk Model and Immune Infiltration Cells

An integrated TCGA immune infiltration data including TIMER, CIBERSORT, XCELL, QUANTISEQ, MCPcounter, EPIC, and CIBERSORT was downloaded from TIMER2.0 (https://timer.comp-genomics.org). The Wilcoxon test was performed to analyze the difference of immune infiltrating status between the high-risk group and low-risk group. Spearman correlation analysis was utilized to analyze the relationship between the immune infiltrating cells and risk score. In above analysis, the R packages of “limma,” “pheatmap,” “scales,” “ggplot2,” “ggtext,” and “ggpubr” were used. Single-sample gene set enrichment analysis (ssGSEA) was utilized to evaluate the difference of immune pathways between the high-risk group and the low-risk group.

### 2.7. Asses the Clinical Significance of the Risk Model in Immune Treatment

Human leukocyte antigen (HLA) and immune check point genes' expression difference between high-risk group and low-risk group were assessed by using “limma,” “reshape2,” “ggplot2,” and “ggpubr” package of R software. In addition, the immunotherapy score data were downloaded from (https://tcia.at/). Tumor immune dysfunction and exclusion prediction score were acquired from (http://tide.dfci.harvard.edu/). The potential sensitivity of high and low-risk group patients to immunotherapy was evaluated to further verify the prognosis function of our risk model.

### 2.8. Statistical Analyses

All data were processed with Perl (5.30.1) or R (version 4.1.0) software. The Wilcoxon rank-sum test was used for differential expressed irlncRNAs. Spearman correlation test and chi-square test were used for the correlation analysis. Survival analyses were performed using the Kaplan-Meier method with log-rank test.

## 3. Results

### 3.1. Identification of Differentially Expressed irlncRNAs in Gastric Cancer

**Figure 1:**
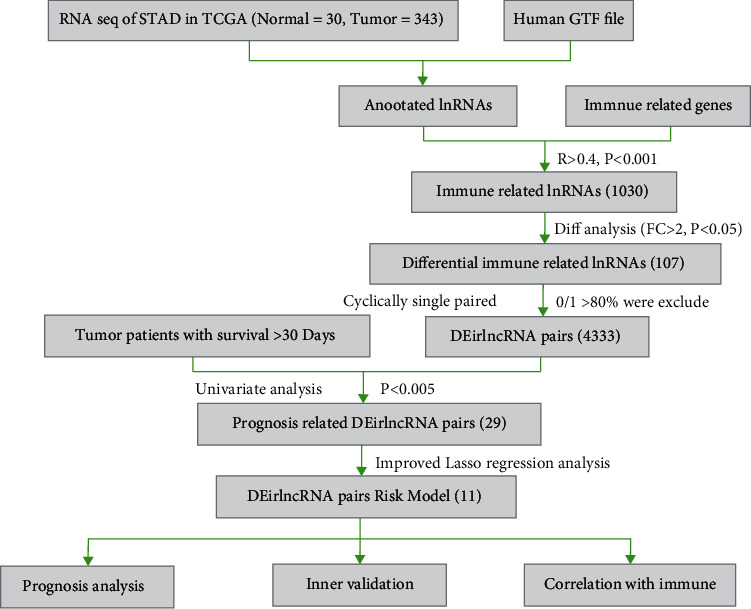
Work flow of this study.

**Figure 2:**
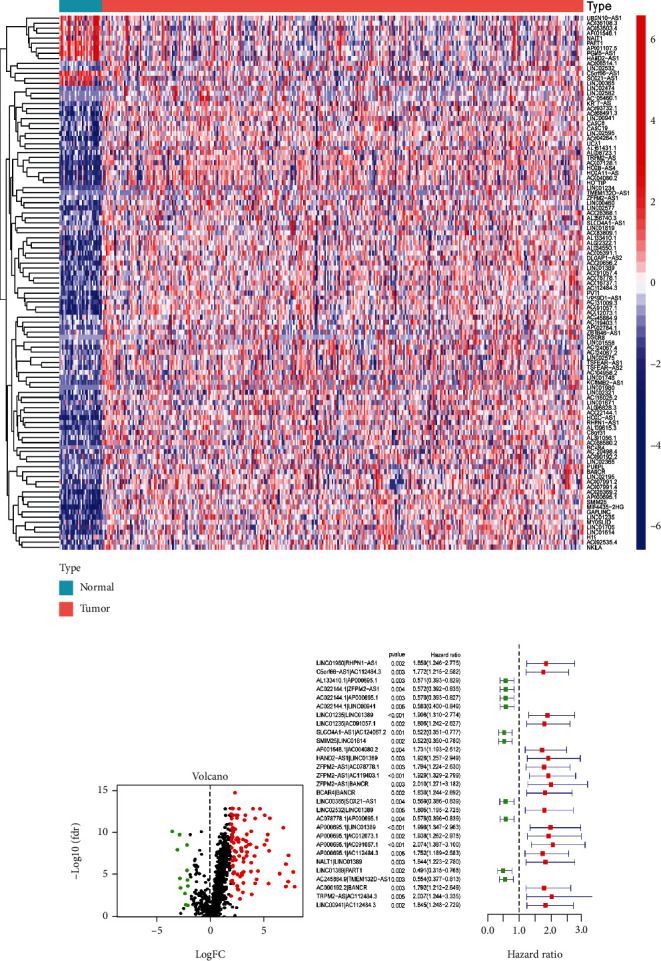
DEirlncRNAs were identified to obtain prognosis-related irlncRNAs. Heatmap was used to visualize the expression of DEirlncRNAs (a). Volcano map was utilized to visualize upregulated and downregulated DEirlncRNAs (b). Univariate analysis was performed to identify prognosis-related DEirlncRNA pairs (c).

The workflow of the present study is summarized in Figure 1. First, we obtained transcription profiling data and corresponding clinical information from The Cancer Genome Atlas (TCGA) database, including 30 normal samples and 343 tumor samples. We then annotated the gene symbols to identify lncRNAs and mRNAs according to the human GTF files. After acquiring the lncRNAs, we performed coexpression analysis between certified immune-related genes and lncRNAs, identifying 1030 immune-related lncRNAs (irlncRNAs). Among these irlncRNAs, 107 were revealed to be differentially expressed irlncRNAs (DEirlncRNAs). Twelve DEirlncRNAs were downregulated, and 95 DEirlncRNAs were upregulated (Figures 2(a) and 2(b)).

### 3.2. Construction of the Risk Model Using DEirlncRNA Pairs

**Figure 3:**
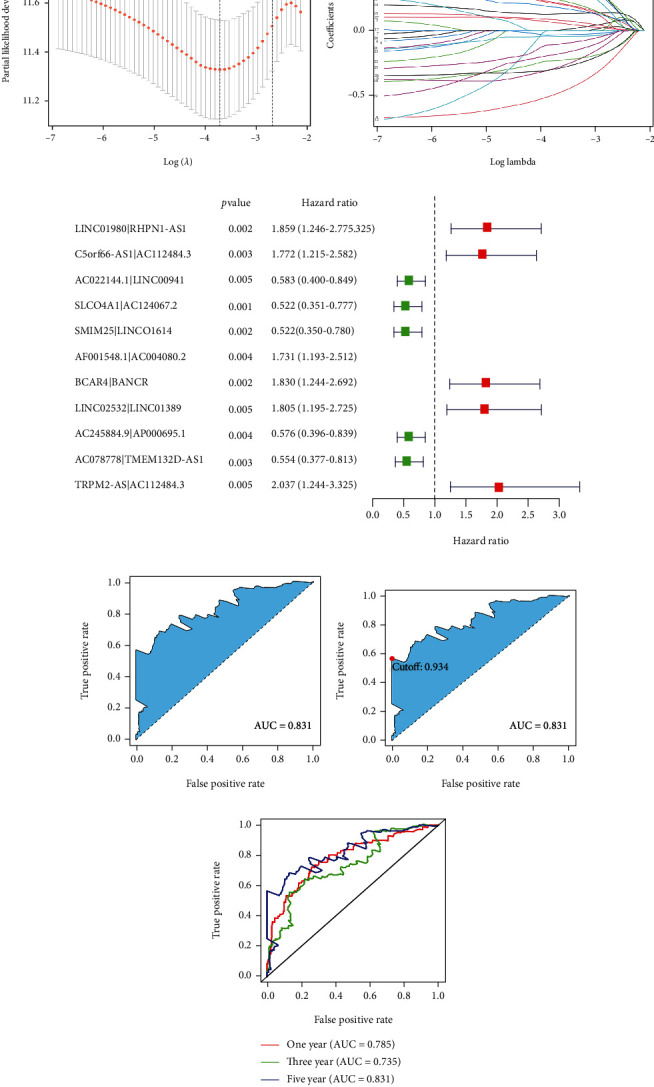
Construction of the risk model. Lasso regression analysis was performed to construct the prognosis ((a) and (b)). A total of 11 DEirlncRNAs pairs were included in the risk model (c). Roc curves were plotted to assess the accuracy of the risk model in predicting patients' survival ((d)–(f)).

The DEirlncRNAs were cyclically paired to construct a new matrix containing DEirlncRNA pairs. A total of 4333 DEirlncRNA pairs were considered valid matches and used for further analysis. After excluding the patients whose survival time was less than 30 days, a total of 305 tumor samples were enrolled to conduct univariate analysis. Twenty-nine DEirlncRNA pairs were identified to be related to the survival of the patients (Figure 2(c)). Next, Lasso regression analysis was performed to determine the DEirlncRNA pairs with the best prognostic value (Figures 3(a) and 3(b)). We obtained a risk model based on 11 DEirlncRNA pairs (Figure 3(c)). A time-dependent receiver operating characteristic (ROC) curve was constructed to verify the accuracy of the risk model. The AUC value confirmed that the identified prognostic model was efficient in predicting the survival of GC patients (Figures 3(d)–3(f)).

**Figure 4:**
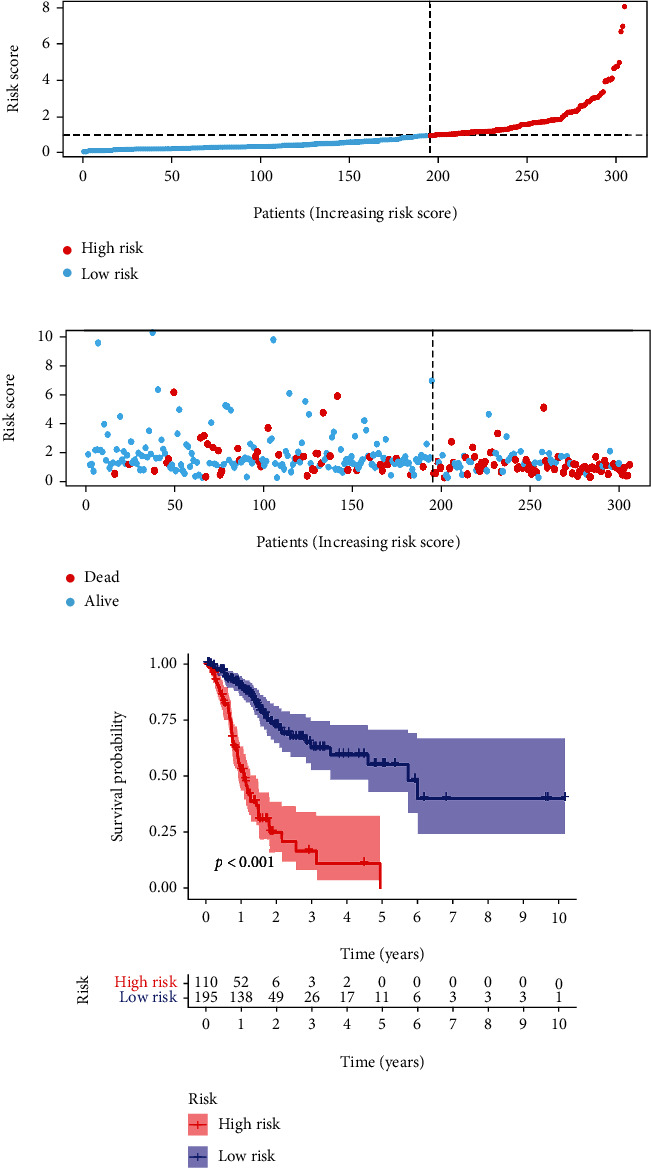
Prognosis of the risk model. Patients were ranked according to the risk score (a). The survival status of the patients in high-risk and low-risk group (b). Kaplan Meier was used to visualize survival probability of the patients in high-risk and low-risk group (c).

According to the risk model, we divided patients into a high-risk group and a low-risk group. We observed that there were more deaths among patients in the high-risk group than those in the low-risk group (Figures 4(a) and 4(b)). In addition, Kaplan-Meier analysis showed that patients in the high-risk group had poorer survival than those in the low-risk group (Figure 4(c)).

### 3.3. Inner Validation of Risk Model

**Figure 5:**
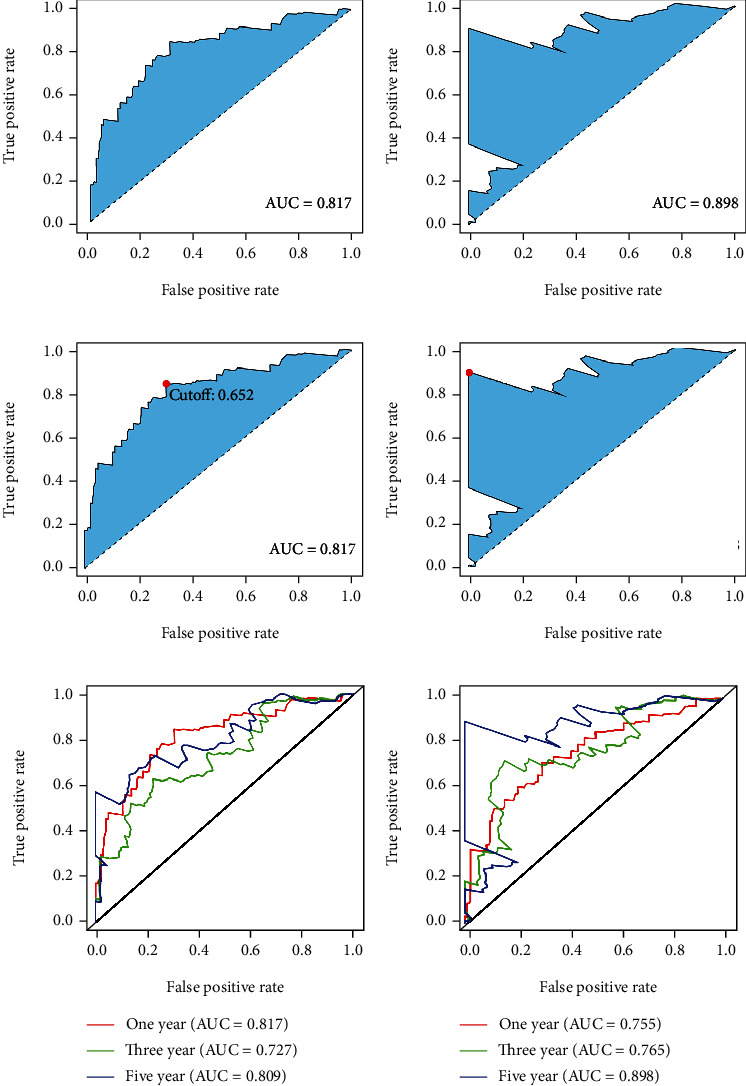
Inner validation of the risk model. Patients were random divided into two validation sets at a ratio of 5 : 5. The maximum AUC values of 5 years exceed over 0.8 in two validation sets ((a) and (b)). The maximum inflection point is the cut-off point ((c) and (d)). One year, three years, and five years AUV value were exhibited, respectively ((e) and (f)).

**Figure 6:**
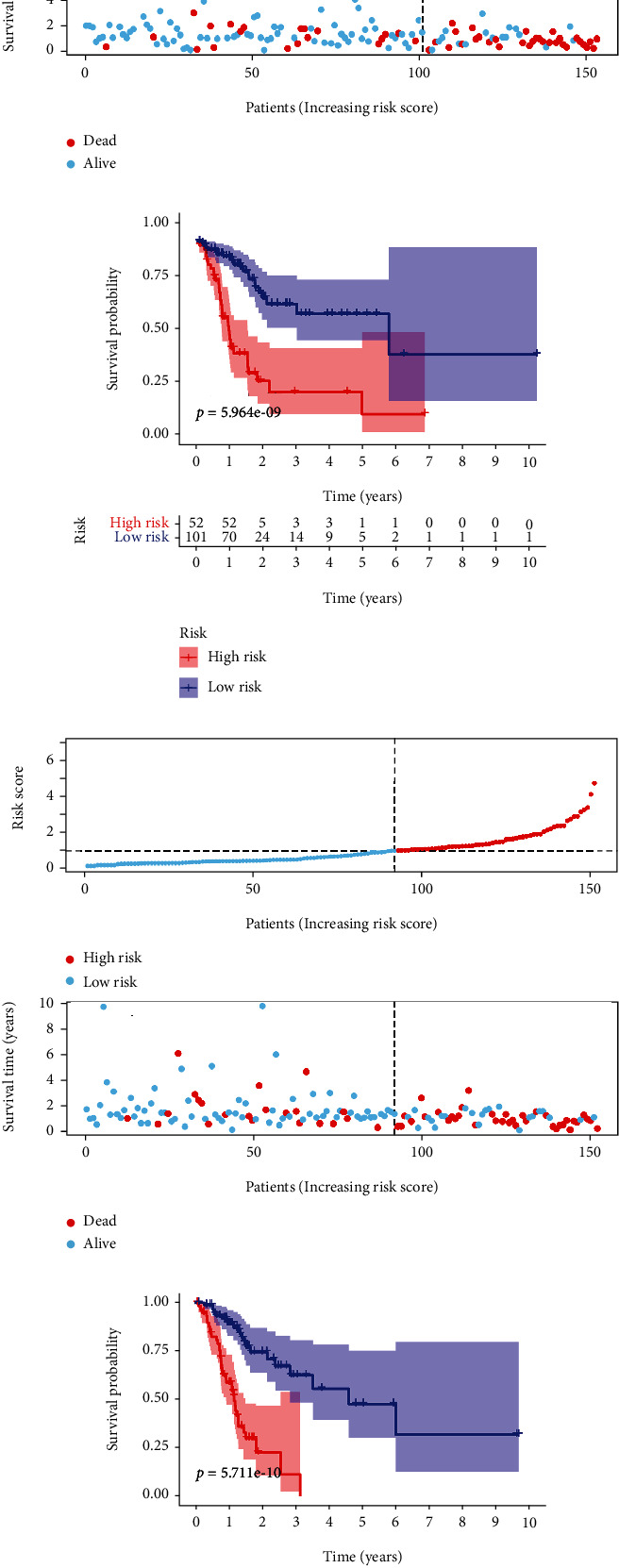
Prognosis prediction of the model in two validation sets. The survival status of the patients in validation set 1 and validation set 2 ((a) and (c)). Kaplan Meier was used to visualize survival probability of the patients in two validation sets ((b) and (d)).

To further validate the predictive efficiency of the risk model, we randomly divided 305 patients into two validation sets as follows: validation set 1 (153 patients) and validation set 2 (152 patients). We then tested the accuracy of the risk model by using a time-dependent ROC curve (Figures 5(a)–5(f)) and found that our risk model had a preferable prognostic performance in the two validation sets. The best AUC values in validation set 1 and validation set 2 were 0.817 and 0.898 (Figures 5(a) and 5(b)), respectively. After sorting the patients according to risk score, we observed that more patients died in the high-risk group than in the low-risk group (Figures 6(a) and 6(c)). Patients with high risk in the two validation sets had a poorer survival probability than low-risk patients (Figures 6(b) and 6(d)). Our results demonstrated that the risk model has reliable predictive performance in patients with GC.

### 3.4. Independent Prognostic Value of the Risk Model

**Figure 7:**
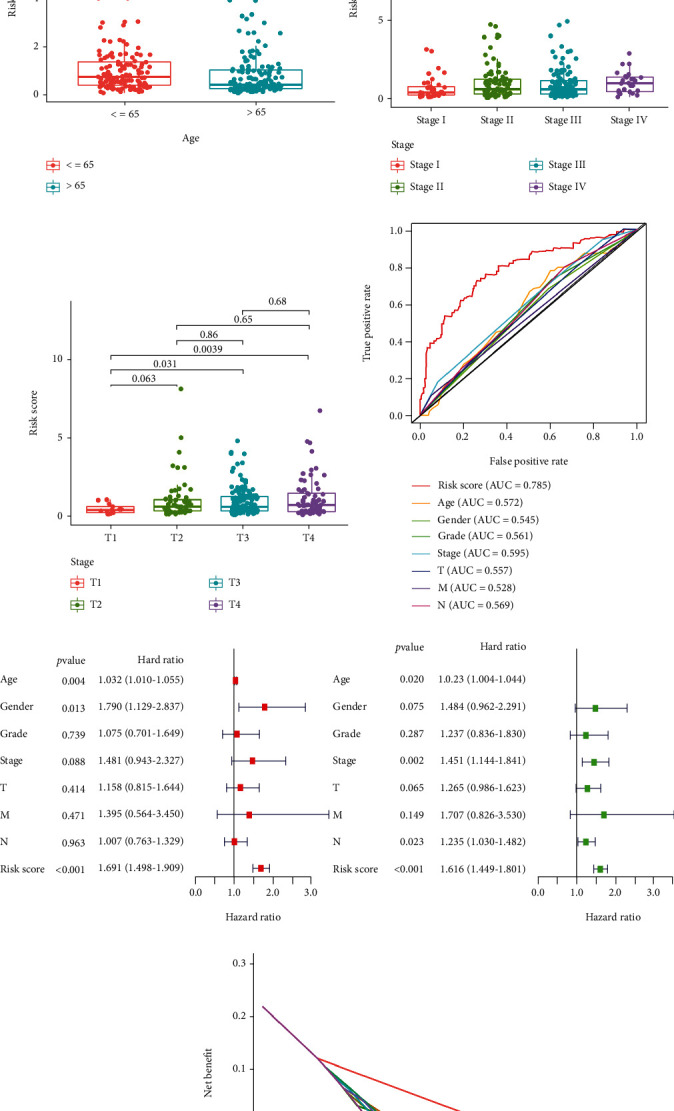
Independent prognosis value of the model. Correlation between the risk score and clinical characteristics (a). Correlation between the risk score and age (b). Correlation between the risk score and tumor stage (c). Correlation between the risk score and T stage (d). ROC curves were plotted to prove the superiority of the risk score (e). Univariate analysis and multivariable analysis were performed to validate the independent prognosis value of the model (f). Decision curve analysis (DCA) was performed to validate the clinical application accuracy of the risk model (g).

To explore the correlation between the risk model and clinical characteristics of the patients, we divided the patients into high- and low-risk groups. We found that our risk model had a positive correlation with patient tumor stage and T stage (Figures 7(a) and 7(c) and 7(d)). In addition, the risk model had a negative correlation with patient age (Figures 7(a) and 7(b)). These results indicated that patients with a higher tumor stage and T stage or with younger age might have poor survival. To further define the independent prognostic value of the risk model, we conducted univariate analysis and multivariable analysis. The results demonstrated that the risk score could be used as an independent prognostic index (Figure 7(f)). ROC curve and decision curve analysis (DCA) were performed to validate the clinical application accuracy of the risk model (Figures 7(e) and 7(g)). These results indicated that our risk model has good performance in independent prognosis.

**Figure 8:**
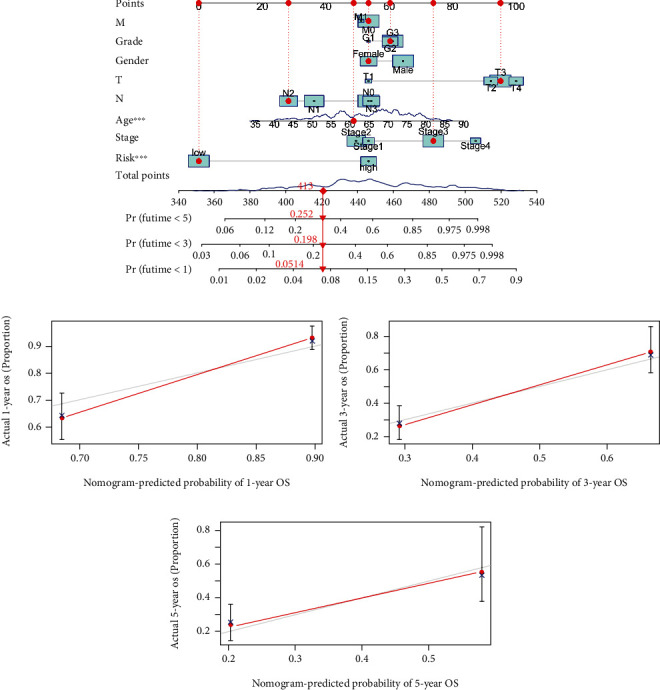
Accuracy validation in prognosis of the risk model. Nomogram was plotted for predicting survival time of patients (a). The accuracy in prognosis of the risk model was determined with calibration curves ((b)–(d)).

To verify the prognosis of our risk model, we constructed a nomogram for predicting the survival time of patients (Figure 8(a)). The calibration curves at one year, three years, and five years were also plotted to assess the accuracy of the nomogram. The predicted survival time of patients at one, three years, and five years was almost consistent with the actual survival time (Figures 8(b)–8(d)), which further proved the accuracy of the risk model.

### 3.5. Association between the Risk Model and Immune Infiltration Cells

**Figure 9:**
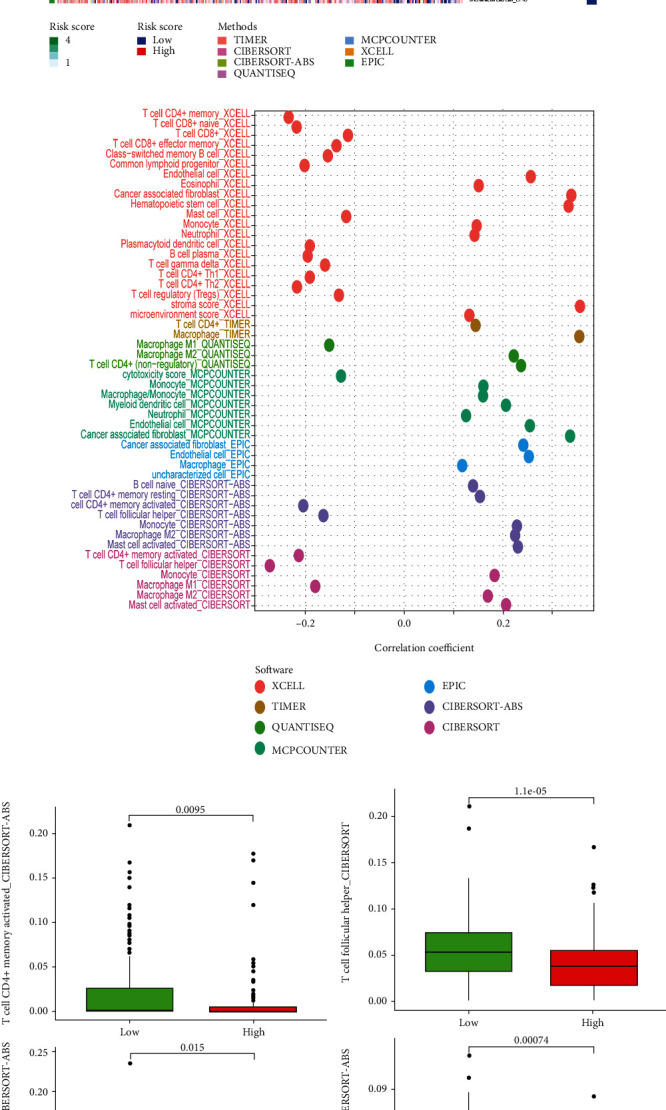
Correlation between risk model and immune infiltration. Immune infiltration status between the high-risk group and the low-risk group was exhibited by heat map and bubble chart ((a) and (b)). Difference in the infiltration status of T cell follicular helper and T cell CD4+ memory activated between the high-risk group and the low-risk group were exhibited ((c) and (d)).

Our risk model was constructed using DEirlncRNA pairs. The status of immune cells in tumors has been reported to be associated with the effectiveness of immunotherapy [29–31]. To better understand whether the prognostic function of our model is related to the tumor immune microenvironment, we obtained the immune infiltration status of GC from TIMER2.0 and compared the difference in infiltrating immune cells between the high-risk group and the low-risk group (Figures 9(a) and 9(b)). The immune infiltration of most T cells was negatively correlated with the risk score (Figure 9(b)), indicating that patients with more T cell infiltration have a lower risk and thus a better prognosis. However, the immune infiltration of most macrophages was positively correlated with the risk score (Figure 9(b)). We also evaluated the infiltration status of T cell follicular helper cells and activated memory CD4+ T cells (Figures 9(c) and 9(d)), and the result agreed with the bubble graph (Figure 9(b)). These results indicated that the risk model can also be used to predict the immune infiltration status of immune cells.

### 3.6. Clinical Significance of the Risk Model in Immune Landscape

**Figure 10:**
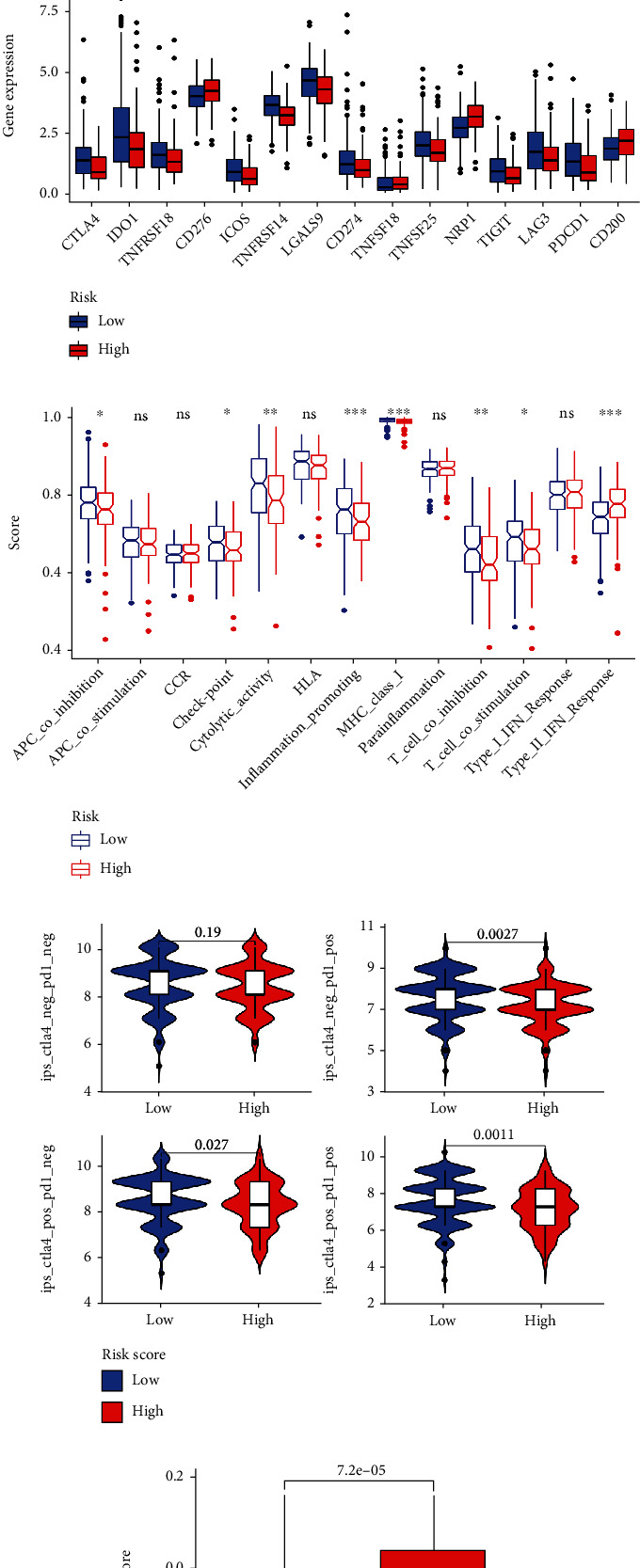
Clinical significance of the risk model in immune landscape. Boxplot was used to visualize the difference of HLA and immune check points between the high-risk group and the low-risk group ((a) and (b)). Enrichment of 13 immune-related pathway was evaluated by using ssGSEA (c). Patients in low-risk group with positive status of CTLA4 or PD-1 have superior immunotherapy scores than high-risk group (d). TIDE prediction score indicated that low-risk patients might be more sensitive to immunotherapy (e).

Then, we evaluated the expression differences of 24 HLA-related genes between low-risk group and high-risk group. Results demonstrated that HLA-F, HLA-DRB5, HLA-L, HLA-E, HLA-H, HLA-DQB1, HLA-J, HLA-DRB1, and HLA-DMA were elevated in low-risk signature group (Figure 10(a)), which indicated that low-risk patients might have a better immune response. The status of the immune checkpoint genes is also associated with the effectiveness of immunotherapy [32], and patients with high expression of immune checkpoint genes might have a better immunotherapy effectiveness [33]. To verify the clinical significance of the risk model, we compared the expression of immune checkpoint genes between high-risk patients and low-risk patients. The results demonstrated that low-risk patients showed a relatively higher expression of most immune checkpoint genes (Figure 10(b)). Furthermore, the ssGSEA method was used to evaluate the difference in enrichment of immune-related pathways between low-risk group and high-risk group. We observed that patients with low-risk exhibited a higher enrichment score of most immune-related pathways (Figure 10(c)). All these results indicated that low-risk group patients might be more sensitive to immunotherapy.

Based on this hypothesis, we downloaded the immunotherapy score data from TCIA (https://tcia.at/) and compared the immunotherapy score between low-risk patients and high-risk patients. We observed that low-risk group patients with single positivity for CTLA4 or PD-1 and double positivity for CTLA4 and PD-1 had higher immunotherapy scores (Figure 10(d)). In addition, we acquired the TIDE prediction score by using tumor immune dysfunction and exclusion website (http://tide.dfci.harvard.edu/). Result also indicated that low-risk group might be more sensitive to immunotherapy (Figure 10(e)). These results confirmed that our risk model has a good performance in predicting the effectiveness of immunotherapy.

## 4. Discussion

Gastric cancer (GC) is a malignant tumor with high mortality that threatens human life and health [2]. Immunotherapies, especially immune checkpoint inhibitors, are new treatments that possess great potential in the treatment of multiple cancers [33–35]. However, patients with GC cancers do not benefit much from immunotherapies compared with chemotherapy and other treatments. Therefore, the identification of effective predictive indicators is urgently needed for GC. Recent studies have shown that long noncoding RNAs (lncRNAs) play crucial roles in tumorigenesis and immune regulation [36, 37]. Many lncRNAs have been identified as signatures of prognosis and immunotherapy in various cancers [27, 38]. Several irlncRNAs have been largely reported to regulate the immune microenvironment or the activation of some immune cells [39, 40]. Investigating the function of irlncRNAs involved in GC is fundamental to the progress of GC immunotherapy. However, current risk models for predicting or evaluating the prognosis of patients with malignant tumors are mainly based on the expression levels of coding and noncoding RNAs [41–43]. Before the application of this type of risk model to other types of gene expression data, the expression data of the genes need to be normalized, which may reduce the accuracy of the model.

In the present study, we established a risk model of immune-related lncRNA (irlncRNA) pairs by a method not based on exact gene expression. First, we obtained the RNA-seq expression profiles of 373 patients from the TCGA database. To identify irlncRNAs, we annotated the RNA-seq matrix using a human GTF file and certified immune-related genes, which resulted in a total of 1030 irlncRNAs. By using the limma package of R software, 107 irlncRNAs were revealed to be differentially expressed between normal tissue and tumor tissue.

For the construction of the risk model, we utilized an improved method of cyclical single pairing along with a 0-or-1 matrix [44], which resulted in 4333 DEirlncRNA pairs. Univariate analysis, multivariate analysis, and lasso penalty regression were conducted to screen the prognosis-related DEirlncRNA pairs. Finally, 11 DEirlncRNA pairs were selected for the construction of the risk model. Some of the DEirlncRNAs (11/22) in the risk model have been previously demonstrated to exert crucial roles in various malignant tumors, including GC, while others were revealed for the first time. For example, linc01980 has been reported to promote esophageal squamous cell carcinoma progression [45]. RHPN1-AS1 has been demonstrated to accelerate the deterioration of gastric cancer and ovarian cancer [46, 47]. C5orf66-AS1 has been defined as a prognostic biomarker in GC and has been shown to promote the proliferation of cervical cancer cells [48, 49]. Linc01614 has also been identified as a prognostic biomarker for GC patients [50]. These findings proposed by other studies further validate that the DEirlncRNAs in our risk model are involved in cancer progression and may be used as prognostic biomarkers.

To test the efficiency of the model, we divided 305 patients into a high-risk group and a low-risk group. As expected, patients in the low-risk cohort had better survival outcomes. A time-dependent receiver operating characteristic (ROC) curve was constructed to validate the accuracy of the risk model. The AUC value of the ROC curve confirmed that our risk model efficiently predicts the survival of GC patients. To further prove the applicability of the model, the total population was randomly divided into two validation sets as follows: validation set 1 (153 patients) and validation set 2 (152 patients). Kaplan-Meier analyses were then conducted, demonstrating that high-risk patients based on the risk model have poor survival probability. The AUC value of the ROC curve exceeded 0.8 in five years. In addition, we performed univariate and multivariate regression analyses and found that our risk model could be an independent prognostic biomarker in predicting patient survival outcomes. Based on the risk model, a nomogram was plotted to obtain the predicted survival probability at one year, three years, and five years, and the results were similar to the actual survival time, further verifying the accuracy of the risk model.

The immune system plays an important role in the development of cancer. Recent studies have demonstrated that immunotherapy prolongs the overall survival (OS) of patients with a variety of cancers [4–7]. The status of immune cell infiltration in tumors is associated with the effectiveness of immunotherapy [29–32]. Patients with more CD4+ and CD8+ T cell infiltration experience a better treatment response from pembrolizumab than those with less infiltration [51, 52]. Infiltration of macrophages in solid tumors is associated with poor prognosis and may enhance tumor progression and metastasis [53]. To explore the relationship between our risk model and the immune landscape, we compared the immune infiltration status of the high-risk group to that of the low-risk group, which demonstrated that patients at high risk had a higher immune infiltration proportion of macrophages but a lower immune infiltration proportion of CD4+ T cells and CD8+ T cells. These results indicated that patients with higher infiltration of CD4+ T cells and CD8+ T cells have a better prognosis and that patients with higher infiltration of macrophages have a poor prognosis, which is consistent with a previous study [51–53]. Human leukocyte antigen- (HLA-) related genes were reported to exert critical function in immune surveillance and response. The human MHC encodes a glycoprotein, HLA, plays a crucial role in T-cell antigen presentation [54]. We observed that patients with lower risk score have a higher expression of most HLA-related genes, indicating a better immune response. Immune checkpoint genes expression level is another indicator of the immune landscape. Patients with higher expression of immune checkpoint genes might have better immunotherapy effectiveness [33]. Our results revealed that patients in the low-risk group had a higher gene expression of various checkpoint genes. Thus, we speculated that low-risk group patients might have a higher immunotherapy score. Subsequently, we acquired immunotherapy score data and tumor immune dysfunction and exclusion prediction score to assess the potential sensitivity of high- and low-risk group patients to immunotherapy. We observed that low-risk patients with double positivity for CTLA4 and PD-1 or single positivity for CTLA4/PD-1 had higher immunotherapy scores. Low-risk group patients have a lower level of TIDE prediction score. These results indicated that our risk model predicts the immune infiltration status and potential sensitivity of immunotherapy in GC patients.

In the present study, a novel prognosis signature constructed by a method not based on exact gene expression was proved to exert an undeniable role in GC. Interestingly, we found that some of the methods in our study are similar to the methods used in another one [26]. However, there were several differences between our study and another one. First, the tumor type is different between two studies. We constructed a novel signature for prognosis predicting and immune landscape in gastric cancer (GC) patients. Another study established a prognostic signature for human hepatocellular carcinoma (HHC) patients. Second, the methods used to validate the function of the signature are quite different. In another study, they only constructed the signature and validated the prognosis function of the signature in the entire patients' samples. They did not perform inner validation. After we obtained the signature in our study, we divided all patients into two subgroups (validation set 1 and validation set 2) and verified prognosis predicting function of our signature in two validation sets, respectively. Third, the author did not determine the clinical application efficiency of their signature in their study. In our study, we constructed a ROC curve and a decision curve analysis (DCA) curve to prove our signature has a better performance in independent prognosis compared with other clinical characteristics. In addition, we also constructed a nomogram for the overall survival predicting in GC patients. We determined that our nomogram could accurately predict survival time of GC patients by using calibration curves. This is another difference between two studies. Finally, the author only detected the immune cells infiltration and expression of immune checkpoint genes as for immune landscape in another study. In our study, we have discussed the function of our signature in immune landscape more depth. Apart from immune cells infiltration and expression of immune checkpoint genes, we determined the difference of 24 HLA-related genes between low-risk group and high-risk group. We also evaluated the difference in enrichment of immune-related pathway between low-risk group and high-risk group by using ssGSEA. In addition, we acquired the immunotherapy score data from TCIA (https://tcia.at/) and TIDE prediction score from tumor immune dysfunction and exclusion website (http://tide.dfci.harvard.edu/) to assess the performance of the signature in predicting the effectiveness of immunotherapy.

Despite the positive findings, the present study has several shortcomings and limitations. The newly developed risk model requires external validation due to the different expression levels of samples in different databases. We failed to acquire data containing mRNA, lncRNA, and clinical data of patients in other databases. Despite the validation in two inner validation sets, external validation from other datasets would be beneficial. Thus, larger samples in multiple centers are needed to verify our results.

## 5. Conclusions

In summary, using DEirlncRNA pairs in GC, we constructed a risk model that accurately predicts the prognosis as well as the immune infiltration status and immunotherapy scores in GC patients. These results provide insights for prognosis prediction of GC patients and important information for immunotherapy in GC.

## Data Availability

All data generated for this study can be found in the (TCGA data portal) (https://cancergenome.nih.gov/).

## References

[1] Sung H., Ferlay J., Siegel R. L. (2021). Global cancer statistics 2020: GLOBOCAN estimates of incidence and mortality worldwide for 36 cancers in 185 countries. *CA: a Cancer Journal for Clinicians*.

[2] Smyth E. C., Nilsson M., Grabsch H. I., van Grieken N. C., Lordick F. (2020). Gastric cancer. *The Lancet*.

[3] Fridman W. H., Zitvogel L., Sautès–Fridman C., Kroemer G. (2017). The immune contexture in cancer prognosis and treatment. *Nature Reviews Clinical Oncology*.

[4] McGranahan N., Furness A. J., Rosenthal R. (2016). Clonal neoantigens elicit T cell immunoreactivity and sensitivity to immune checkpoint blockade. *Science (New York, N.Y.)*.

[5] Jin T., Zhang Q., Jin Q. F., Hua Y. H., Chen X. Z. (2021). Anti-PD1 checkpoint inhibitor with or without chemotherapy for patients with recurrent and metastatic nasopharyngeal carcinoma. *Translational Oncology*.

[6] Herbst R. S., Baas P., Kim D. W. (2016). Pembrolizumab versus docetaxel for previously treated, PD-L1-positive, advanced non-small-cell lung cancer (KEYNOTE-010): a randomised controlled trial. *The Lancet*.

[7] Donato E. M., Fernández-Zarzoso M., De La Rubia J. (2017). Immunotherapy for the treatment of Hodgkin lymphoma. *Expert Review of Hematology*.

[8] Zhao Q., Cao L., Guan L. (2019). Immunotherapy for gastric cancer: dilemmas and prospect. *Briefings in Functional Genomics*.

[9] Marrelli D., Polom K., Pascale V. (2016). Strong prognostic value of microsatellite instability in intestinal type non-cardia gastric cancer. *Annals of Surgical Oncology*.

[10] Li B., Chan H. L., Chen P. (2019). Immune checkpoint inhibitors: basics and challenges. *Current Medicinal Chemistry*.

[11] Ralph C., Elkord E., Burt D. J. (2010). Modulation of lymphocyte regulation for cancer therapy: a phase II trial of tremelimumab in advanced gastric and esophageal adenocarcinoma. *Clinical Cancer Research : an official journal of the American Association for Cancer Research*.

[12] Kang Y. K., Boku N., Satoh T. (2017). Nivolumab in patients with advanced gastric or gastro-oesophageal junction cancer refractory to, or intolerant of, at least two previous chemotherapy regimens (ONO-4538-12, ATTRACTION-2): a randomised, double-blind, placebo- controlled, phase 3 trial. *The Lancet*.

[13] Fuchs C. S., Doi T., Jang R. W. (2018). Safety and efficacy of pembrolizumab monotherapy in patients with previously treated advanced gastric and gastroesophageal junction Cancer. *JAMA Oncology*.

[14] Shitara K., Özgüroğlu M., Bang Y. J. (2018). Pembrolizumab versus paclitaxel for previously treated, advanced gastric or gastro-oesophageal junction cancer (KEYNOTE-061): a randomised, open-label, controlled, phase 3 trial. *The Lancet*.

[15] Chi Y., Wang D., Wang J., Yu W., Yang J. (2019). Long non-coding RNA in the pathogenesis of cancers. *Cell*.

[16] Palazzo A. F., Lee E. S. (2015). Non-coding RNA: what is functional and what is junk?. *Frontiers in Genetics*.

[17] Zhang Z., Wan J., Liu X., Zhang W. (2020). Strategies and technologies for exploring long noncoding RNAs in heart failure. *Biomedicine & Pharmacotherapy*.

[18] Bhan A., Soleimani M., Mandal S. S. (2017). Long noncoding RNA and cancer: a new paradigm. *Cancer Research*.

[19] Peng W. X., Koirala P., Mo Y. Y. (2017). LncRNA-mediated regulation of cell signaling in cancer. *Oncogene*.

[20] Fattahi S., Kosari-Monfared M., Golpour M. (2020). LncRNAs as potential diagnostic and prognostic biomarkers in gastric cancer: a novel approach to personalized medicine. *Journal of Cellular Physiology*.

[21] Chen L., Zhang Y. H., Lu G., Huang T., Cai Y. D. (2017). Analysis of cancer-related lncRNAs using gene ontology and KEGG pathways. *Artificial Intelligence in Medicine*.

[22] Xu F., Huang X., Li Y., Chen Y., Lin L. (2021). m^6^A-related lncRNAs are potential biomarkers for predicting prognoses and immune responses in patients with LUAD. *Molecular Therapy Nucleic Acids*.

[23] Dastmalchi N., Safaralizadeh R., Nargesi M. M. (2020). LncRNAs: potential novel prognostic and diagnostic biomarkers in colorectal cancer. *Current Medicinal Chemistry*.

[24] Wang L., Cho K. B., Li Y., Tao G., Xie Z., Guo B. (2019). Long noncoding RNA (lncRNA)-mediated competing endogenous RNA networks provide novel potential biomarkers and therapeutic targets for colorectal cancer. *International Journal of Molecular Sciences*.

[25] Yu W. D., Wang H., He Q. F., Xu Y., Wang X. C. (2018). Long noncoding RNAs in cancer-immunity cycle. *Journal of Cellular Physiology*.

[26] Hong W., Liang L., Gu Y. (2020). Immune-related lncRNA to construct novel signature and predict the immune landscape of human hepatocellular carcinoma. *Molecular Therapy Nucleic Acids*.

[27] Shen Y., Peng X., Shen C. (2020). Identification and validation of immune-related lncRNA prognostic signature for breast cancer. *Genomics*.

[28] Tanaka A., Sakaguchi S. (2017). Regulatory T cells in cancer immunotherapy. *Cell Research*.

[29] Zhang J., Endres S., Kobold S. (2019). Enhancing tumor T cell infiltration to enable cancer immunotherapy. *Immunotherapy*.

[30] Anfray C., Ummarino A., Andón F. T., Allavena P. (2020). Current strategies to target tumor-associated-macrophages to improve anti-tumor immune responses. *Cell*.

[31] Burugu S., Dancsok A. R., Nielsen T. O. (2018). Emerging targets in cancer immunotherapy. *Seminars in Cancer Biology*.

[32] Sharma P., Siddiqui B. A., Anandhan S. (2021). The next decade of immune checkpoint therapy. *Cancer Discovery*.

[33] Barrios D. M., Do M. H., Phillips G. S. (2020). Immune checkpoint inhibitors to treat cutaneous malignancies. *Journal of the American Academy of Dermatology*.

[34] Himmel M. E., Saibil S. D., Saltman A. P. (2020). Immune checkpoint inhibitors in cancer immunotherapy. *CMAJ : Canadian Medical Association Journal = journal de l'Association medicale canadienne*.

[35] Nie K., Zheng Z., Wen Y. (2020). A novel ceRNA axis involves in regulating immune infiltrates and macrophage polarization in gastric cancer. *International Immunopharmacology*.

[36] Wu Y., Zhang L., He S. (2020). Identification of immune-related lncRNA for predicting prognosis and immunotherapeutic response in bladder cancer. *Aging*.

[37] Zhang M., Wang N., Song P. (2020). LncRNA GATA3-AS1 facilitates tumour progression and immune escape in triple-negative breast cancer through destabilization of GATA3 but stabilization of PD-L1. *Cell Proliferation*.

[38] Jin D., Song Y., Chen Y., Zhang P. (2020). Identification of a seven-lncRNA immune risk signature and construction of a predictive nomogram for lung adenocarcinoma. *BioMed Research International*.

[39] Li J., Zhang C., Zhang C., Wang H. (2020). Construction of immune-related and prognostic lncRNA clusters and identification of their immune and genomic alterations characteristics in lung adenocarcinoma samples. *Aging*.

[40] Zhou M., Zhao H., Xu W., Bao S., Cheng L., Sun J. (2017). Discovery and validation of immune-associated long non-coding RNA biomarkers associated with clinically molecular subtype and prognosis in diffuse large B cell lymphoma. *Molecular Cancer*.

[41] Liu Z., Zhong J., Cai C., Lu J., Wu W., Zeng G. (2020). Immune-related biomarker risk score predicts prognosis in prostate cancer. *Aging*.

[42] Milanez-Almeida P., Martins A. J., Germain R. N., Tsang J. S. (2020). Cancer prognosis with shallow tumor RNA sequencing. *Nature Medicine*.

[43] Luo M. S., Huang G. J., Liu H. B. (2020). An autophagy-related model of 4 key genes for predicting prognosis of patients with laryngeal cancer. *Medicine*.

[44] Sveen A., Ågesen T. H., Nesbakken A. (2012). ColoGuidePro: a prognostic 7-gene expression signature for stage III colorectal cancer patients. *Clinical Cancer Research : an official journal of the American Association for Cancer Research*.

[45] Liang X., Wu Z., Shen S. (2020). LINC01980 facilitates esophageal squamous cell carcinoma progression via regulation of miR-190a-5p/MYO5A pathway. *Archives of Biochemistry and Biophysics*.

[46] Ding L., Wang L., Li Z., Jiang X., Xu Y., Han N. (2020). The positive feedback loop of RHPN1-AS1/miR-1299/ETS1 accelerates the deterioration of gastric cancer. *Biomedicine & Pharmacotherapy*.

[47] Zhao L., Liu T., Zhang X., Zuo D., Liu C. (2020). lncRNA RHPN1-AS1 promotes ovarian cancer growth and invasiveness through inhibiting miR-1299. *Oncotargets and Therapy*.

[48] Zhou Q., Li H., Jing J., Yuan Y., Sun L. (2020). Evaluation of C5orf66-AS1 as a potential biomarker for predicting early gastric cancer and its role in gastric Carcinogenesis. *Oncotargets and Therapy*.

[49] Rui X., Xu Y., Jiang X., Ye W., Huang Y., Jiang J. (2018). Long non-coding RNA C5orf66-AS1 promotes cell proliferation in cervical cancer by targeting miR-637/RING1 axis. *Cell Death & Disease*.

[50] Chen Y., Cheng W. Y., Shi H. (2021). Classifying gastric cancer using FLORA reveals clinically relevant molecular subtypes and highlights *LINC01614* as a biomarker for patient prognosis. *Oncogene*.

[51] Zander R., Schauder D., Xin G. (2019). CD4^+^ T Cell Help Is Required for the Formation of a Cytolytic CD8^+^ T Cell Subset that Protects against Chronic Infection and Cancer. *Immunity*.

[52] Cassetta L., Pollard J. W. (2018). Targeting macrophages: therapeutic approaches in cancer. *Nature Reviews Drug Discovery*.

[53] Qian B. Z., Pollard J. W. (2010). Macrophage diversity enhances tumor progression and metastasis. *Cell*.

[54] Mosaad Y. M. (2015). Clinical role of human leukocyte antigen in health and disease. *Scandinavian Journal of Immunology*.

